# Genetic Characterization of Japanese Encephalitis Virus Genotype 5 Isolated from Patient, South Korea, 2015

**DOI:** 10.3201/eid2605.190977

**Published:** 2020-05

**Authors:** Jae Hoon Woo, Young Eui Jeong, Jung Eun Jo, Sang-Mu Shim, Jungsang Ryou, Kyung-Chang Kim, Won Ja Lee, Joo-Yeon Lee

**Affiliations:** Centers for Disease Control and Prevention, Chongju, South Korea (J.H. Woo, Y.E. Jeong, J.E. Jo, S.-M. Shim, J. Ryou, K.-C. Kim, W.J. Lee, J.-Y. Lee);; MIRIMEDIX Corporation, Gangwon, South Korea (Y.E. Jeong)

**Keywords:** Japanese encephalitis virus, viruses, genotype 5, genetic characterization, South Korea

## Abstract

We isolated Japanese encephalitis virus genotype 5 from human specimens in South Korea. Whole-genome analysis showed 90.4% identity with other genotype 5 viruses from humans. This virus had a unique insertion in the NS4A gene. However, the envelope protein contained Lys 84, which was specific to strains of genotype 5 viruses from South Korea.

Japanese encephalitis is caused by Japanese encephalitis virus (JEV), a mosquitoborne virus of the family *Flaviviridae*, genus *Flavivirus* (*1*). The JEV genome is composed of a single-stranded, positive-sense RNA of ≈11 kb with a single open reading frame (ORF) encoding a polyprotein. The polyprotein is processed into 3 structural proteins, capsid, membrane, and envelope (E), and 7 nonstructural proteins, NS1, NS2A, NS2B, NS3, NS4A, NS4B, and NS5 (*2*).

JEV is distributed in temperate and tropical areas of eastern and Southeast Asia. In 2010, JEV genotype 1 was the predominant virus circulating. However, genotype 5 was also identified in mosquitoes in South Korea (*3*). Since that time, JEV genotype 5 has been detected in mosquitoes in many areas of South Korea (*4*). We report isolation of JEV genotype 5 virus from patient specimens and differences in sequences among other JEV strains (genotypes 1–5).

## The Study

We isolated JEV (strain K15P38) from samples of a 27-year-old woman who came to a hospital in Kyeonggi-do, South Korea, on November 8, 2015. The patient had mild symptoms, such as fever, headache, apathy, and nausea. The patient recovered. We obtained documentation from the hospital that she had been vaccinated against Japanese encephalitis. Cerebrospinal fluid (CSF) and serum samples were obtained during the acute and convalescent phases.

We detected JEV IgM in serum and CSF samples by using an ELISA (Inbios, https://inbios.com) for convalescent-phase samples, but not acute-phase samples. We isolated virus by inoculating the convalescent-phase CSF sample onto BHK-21 cells. After a cytopathic effect was observed, we confirmed presence of virus by using a quantitative real-time PCR. We performed whole-genome sequence analysis of the virus by using virus genome extracted from 5 passaged culture supernatants and QIAamp Viral RNA Mini Kit (QIAGEN, https://www.qiagen.com).

We performed next-generation sequencing for full-length genes by using the Illumina (https://www.illumina.com) and confirmed gaps from next-generation sequencing by using Sanger sequencing. We assembled nucleotide sequences by using the SeqMan program in DNASTAR software version 5.06 (https://www.dnastar.com). We then conducted molecular phylogenetic analysis of ORF nucleotide sequences with 30 previously reported JEV strains by using MEGA 6.0 software (https://www.megasoftware.net) and the maximum-likelihood method (*5*) and calculated time-scale phylogenies by using BEAST version 2.6.0 software (*6*). We deposited the polyprotein genome sequence of strain K15P38 in GenBank (accession no. MK541529).

**Figure 1:**
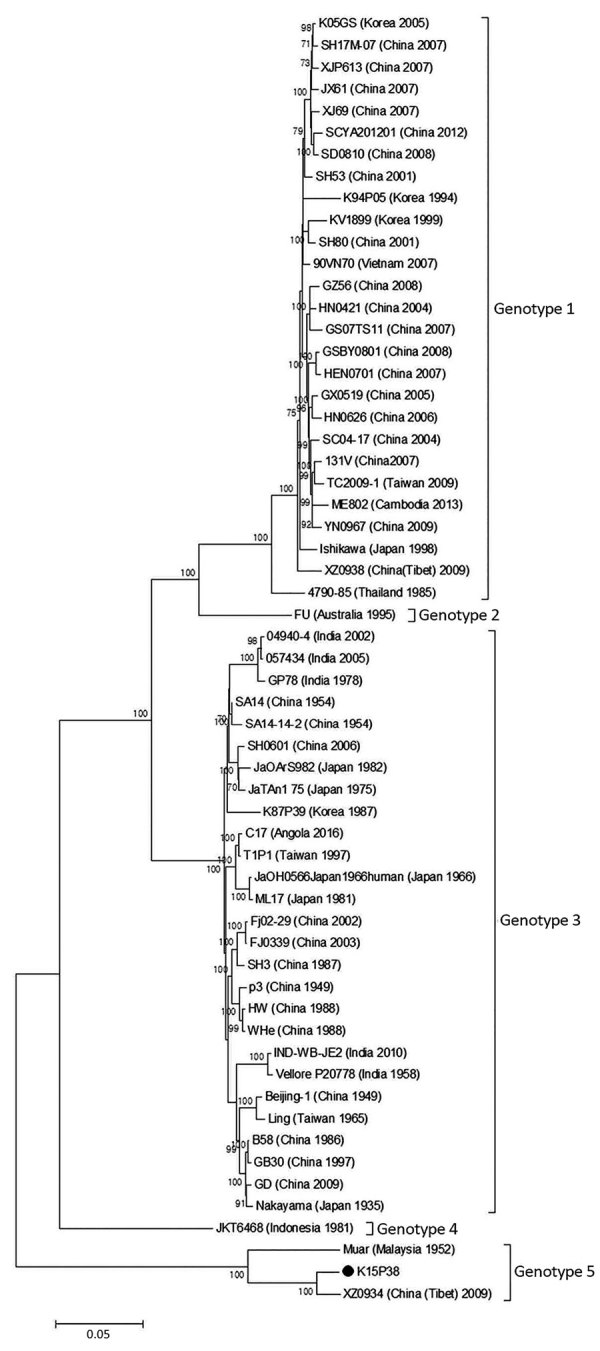
Phylogenetic tree of Japanese encephalitis virus genotypes 1–5, South Korea. Entire open reading frame is shown. Bootstrap probabilities (values along branches) of each node were calculated by using 1,000 replicates. Branches showing quartet puzzling reliability >70% can be considered well supported. Black circle indicates K15P38 strain from patient samples. Scale bar indicates nucleotide substitutions per site.

**Figure 2:**
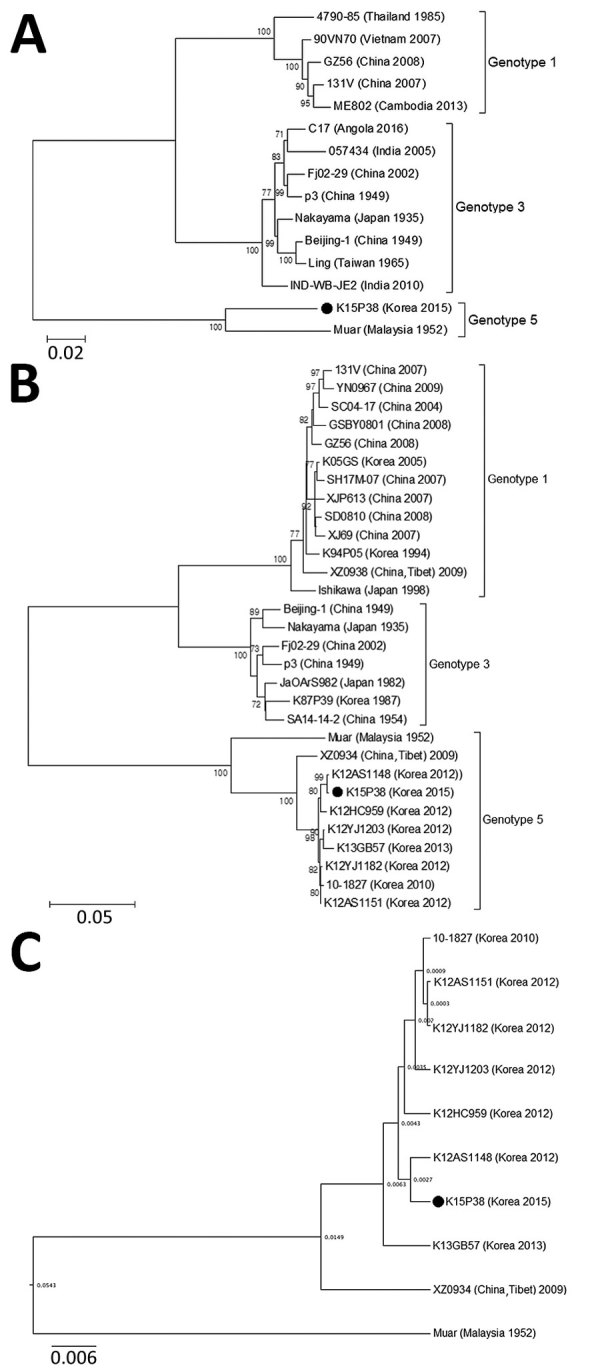
Phylogenetic trees of Japanese encephalitis virus (JEV) genotypes 1, 3, and 5, South Korea. A) Entire open reading frame of JEV human isolates. B) Envelope protein genes of JEV human isolates. C) Divergence time estimation based on the envelope protein genes of JEV genotype 5. Bootstrap probabilities (values along branches) of each node were calculated by using 1,000 replicates. Branches showing quartet puzzling reliability >70% can be considered well supported. Black circles indicate K15P38 strain from patient samples. Scale bars indicate nucleotide substitutions per site.

We compared the entire ORF sequences of K15P38 virus with previously reported strains of JEV genotypes 1–5. Phylogenetic analysis showed that K15P38 belonged to JEV genotype 5 by (Figures 1, 2, panel A; Table 1). Identities between the entire ORF of K15P38 and Muar genotype 5 virus were 90.4%.

**Table 1:** Data on 67 JEV strains analyzed in study of JEV in patient samples from South Korea*

Strain	GenBank accession no.	Country	Year	Genotype	Host
ME802	KY927819	Cambodia	2013	1	Human
XJ69	EU880214	China	2007	1	Mosquito
YN0967	JF706268	China	2009	1	Mosquito
SH53	JN381850	China	2001	1	NA
SD0810	JF706286	China	2008	1	Mosquito
HN0626	JN381837	China	2006	1	NA
HN0421	JN381841	China	2004	1	NA
GX0519	JN381835	China	2005	1	NA
131V	GU205163	China	2007	1	Human
GSBY0801	JF706274	China	2008	1	Mosquito
GS07TS11	JN381843	China	2007	1	NA
XJP613	EU693899	China	2007	1	Mosquito
SH80	JN381848	China	2001	1	NA
SH17M-07	EU429297	China	2007	1	Mosquito
SCYA201201	KM658163	China	2012	1	Swine
SC04–17	GU187972	China	2004	1	Mosquito
JX61	GU556217	China	2008	1	Swine
HEN0701	FJ495189	China	2007	1	Swine
GZ56	HM366552	China	2008	1	Human
XZ0938	HQ652538	China (Tibet)	2009	1	Mosquito
Ishikawa	AB051292	Japan	1998	1	Mosquito
KV1899	AY316157	South Korea	1999	1	NA
K94P05	AF04551	South Korea	1994	1	Mosquito
K05GS	KR908702	South Korea	2005	1	Mosquito
TC2009–1	JF499790	Taiwan	2009	1	Mosquito
4790–85	GQ902062	Thailand	1985	1	Human
90VN70	HM228921	Vietnam	1990	1	Human
FU	AF217620	Australia	1995	2	NA
C17	KX945367	Angola	2016	3	Human
SH3	JN381864	China	1987	3	NA
P3	U47032	China	1949	3	Human
GD	JN711458	China	2009	3	Bat
FJ0339	JN381859	China	2003	3	NA
Fj02–29	JF706273	China	2002	3	Human
Beijing-1	L48961	China	1949	3	Human
SA14	KU323483	China	1954	3	NA
SA14–14–2	AF315119	China	1954	3	Vaccine stain
B58	FJ185036	China	1986	3	Bat
HW	AY849939	China	1988	3	NA
Whe	EF107523	China	1988	3	Swine
GB30	FJ185037	China	1997	3	Bat
SH0601	EF543861	China	2006	3	NA
Vellore P20778	AF080251	India	1958	3	NA
04940–4	EF623989	India	2002	3	Mosquito
IND-WB-JE2	JX072965	India	2010	3	Human
GP78	AF075723	India	1978	3	NA
57434	EF623988	India	2005	3	Human
Nakayama	EF571853	Japan	1935	3	Human
JaOArS982	M18370	Japan	1982	3	Mosquito
JaOH0566/Japan/1966/human	AY508813	Japan	1966	3	NA
JaTAn1/75	AB551990	Japan	1975	3	Swine
ML17	AY508812	Japan	1981	3	NA
K87P39	AY585242	South Korea	1987	3	Mosquito
T1P1	AF254453	Taiwan	1997	3	Mosquito
Ling	L78128	Taiwan	1965	3	Human
JKT6468	AY184212	Indonesia	1981	4	Mosquito
XZ0934	JF915894	China (Tibet)	2009	5	Mosquito
Muar	HM596272	Malaysia	1952	5	Human
10–1827	JN587258	South Korea	2010	5	Mosquito
K12HC959	KJ420589	South Korea	2012	5	Mosquito
K12AS1148	KJ420590	South Korea	2012	5	Mosquito
K12AS1151	KJ420591	South Korea	2012	5	Mosquito
K12YJ1174	KJ420593	South Korea	2012	5	Mosquito
K12YJ1182	KJ420594	South Korea	2012	5	Mosquito
K12YJ1203	KJ420592	South Korea	2012	5	Mosquito
K13GB57	KM496503	South Korea	2013	5	Mosquito

*JEV, Japanese encephalitis virus; NA, not available.

In general, the E gene of JEV plays a major role in the pathogenesis of encephalitis (*7*). Several amino acids, including 107, 138, and 176 in the E protein, are reported to play major roles in the neurovirulence of JEV. K15P38 virus had conserved amino acids at these sites (*8**,**9*). However, the E protein of this virus had 6 different amino acids compared with that of the Muar strain isolated from a human in Malaysia in 1952 (*10*). Because Muar virus was derived from mouse brain and K15P38 virus was passaged in cell culture, we do not exclude the possibility of sequence variation caused by different culture methods.

Furthermore, the K15P38 strain contained Lys rather than Arg at position 84 of the E protein (Table 2), which was unique in genotype 5 viruses from South Korea strains derived from mosquito and human specimens. The E gene sequences of K15P38 virus showed high identity of ≈98.5%–99.8% with other genotype 5 strains from South Korea isolated from mosquitoes (Figure 2, panel B). By estimating the root of the time measured on the E gene of JEV genotype 5 viruses, we identified that the XZ0934 strain from Tibet was an ancestor of JEV genotype 5 virus strains from South Korea (Figure 2, panel C). Considering these variations and time estimation of JEV genotype 5, further study is needed to investigate molecular and biologic characteristics of JEV.

**Table 2:** Comparison of amino acid sequences of envelope protein of Japanese encephalitis viruses of genotype 5, South Korea*

Virus	Amino acid position												
	42	52	58	84	129	156	161	171	208	240	292	343	473
K15P38 (South Korea 2015)	D	E	T	K	I	T	A	P	S	M	E	A	I
10-1827 (South Korea 2010)	.	.	.	.	.	.	.	.	.	.	.	.	.
K12AS1148 (South Korea 2012)	.	.	.	.	.	.	.	.	.	.	.	.	.
K12AS1151 (South Korea 2012)	.	.	.	.	.	.	.	.	.	.	.	.	.
K12HC95 (South Korea 2012)	.	.	A	.	T	.	.	.	.	.	.	V	.
K12YJ1182 (South Korea 2012)	.	.	.	.	.	.	.	.	.	.	.	.	.
K12YJ1203 (South Korea 2012)	.	.	.	.	.	.	.	L	.	.	.	.	.
K13GB57 (South Korea 2013)	G	.	.	.	.	.	V	.	.	.	.	.	T
Muar (Malaysia 1952)	.	Q	.	R	.	S	.	.	T	L	D	.	.
XZ0934 (China (Tibet) 2009)	.	.	.	R	.	.	.	.	.	.	.	.	.

*Dots indicate 100% amino acid sequence identity.

## Conclusions

JEV genotype 5 was isolated from mosquitoes in China during 2009 and South Korea during 2010. Because the major JEV genotype from mosquitoes in South Korea changed from genotype 1 to genotype 5 during 2010, the number of infected patients increased coincidently, especially adult patients (*8**,**11*). Japanese encephalitis is generally more prevalent in southern areas of South Korea, wherein *Culex tritaeniorhynchus* mosquitoes are more prevalent than in other regions. However, the prevalence of Japanese encephalitis has also increased in northern regions of South Korea, including Seoul, Gyeonggi, and Gangwon since 2010. This finding is consistent with the fact that 5 JEVs with genotype 5 have been reported in more diverse mosquito species, including *Cx. orientalis* and *Cx pipiens*, not only in *Cx*. *tritaeniorincus* (*4*).

A previous study showed that the prevalence of neutralizing antibodies to JEV were maintained at a level of 98.1% among the general population (*12*) because of the National Vaccine Program against Japanese encephalitis in South Korea since 1982. The currently used Japanese encephalitis vaccine that contains the JEV genotype 3 strain provides adequate protection against JEV genotype 1 (*13*).

Even so, the number of adult patients with Japanese encephalitis has increased. It has also been reported that existing JEV genotype 3 vaccines are less effective in protecting against JEV genotype 5 (*14*), suggesting the need for studies of the protective effect of current Japanese encephalitis vaccine against JEV genotype 5 virus.

Although JEV genotype 5 is highly pathogenic and causes early viremia and central nervous system invasion in animal models, limited information is available on the biological nature of JEV G5. Our results provide potentially useful information regarding JEV genotype 5, including pathogenic characteristics and vaccine efficacy.
